# Utilization of palm residues for biochar production using continuous flow pyrolysis unit

**DOI:** 10.1016/j.fochx.2023.100903

**Published:** 2023-09-28

**Authors:** Mahmoud Younis, Hesham A. Farag, Abdulla Alhamdan, Galal Aboelasaad, Assem I. Zein El-Abedein, Reham M. Kamel

**Affiliations:** a Chair of Dates Industry and Technology, Department of Agricultural Engineering, College of Food and Agricultural Sciences, King Saud University, PO Box 2460, Riyadh 11451, Saudi Arabia; b Agricultural Engineering Research Institute, Agricultural Research Center, Giza 12611, Egypt; c Department of Agricultural Engineering, College of Food and Agricultural Sciences, King Saud University, Riyadh 11451, Saudi Arabia

**Keywords:** Date palm fronds, Biochar, Pyrolysis, Continuous flow, Physical and chemical properties

## Abstract

Biochar is a carbonaceous solid substance produced by heating biomass without using air. This research aimed to create and evaluate local carbonization pyrolysis using a screw conveyor and filtration equipment. Date palm frond (DPF) biochar was studied and tested at pyrolysis temperatures of 320, 390, and 460 °C, as well as feeding rates of 60, 90, and 120 kg/h. The physicochemical parameters of DPF biochar were evaluated using SEM and FTIR. When the pyrolysis temperature was raised from 320 to 450 °C, and the feed rates were reduced from 120 to 60 kg/h, the biochar yield of DPF and volatiles fell. At 460 °C and 60 kg/h, the maximum ash and fixed carbon content were 11.73 and 77.61 %, respectively. As the feed rate decreased and the temperature increased, the H and O values (1.96 and 2.62 %, respectively) of DPF biochar decreased considerably; the C and N values (83.60 and 0.24 %, respectively) trended in opposite directions. The BET surface area and pore volume increased as a result of the micropore surface area and volume at higher temperatures and lower feeding rates, but water holding capacity increased from 6.04 g_water_/10 g at 320 °C to 6.78 g_water_/10 g at 390 °C (60 kg/h). The results showed that the heating temperature increased and the feeding rate decreased; phosphorus) P(and magnesium (Mg) increased significantly, whereas the levels of potassium (K) and calcium (Ca) showed a non-significant increase. Furthermore, as the pyrolysis temperature increased, pH and EC increased from 7.90 to 10.96 and 2.91 to 4.25 dSm^−1^, respectively, while CEC declined; however, there were no significant changes in CEC. DPF biochar demonstrated enhanced macro porosity and surface area at 460 °C and 60 kg/h, making it acceptable for agricultural use as a soil supplement.

## Highlights

• Date palm frond biochar development and evaluation utilizing a screw conveyor and filtration unit.
• Enhance physical and chemical properties with increased pyrolysis temperature and decreased feeding rate.
• At 460 °C and 60 kg/h, date palm fronds biochar had enhanced macro porosity and surface area.

## Introduction

The rapid expansion of the global population has resulted in a heightened need for agricultural commodities, causing a surplus of agricultural waste in landfills and bodies of water. The combustion process results in the emission of substantial quantities of smoke and soot, leading to the depletion of essential nutrients and microbial communities within the soil. In addition, this practice contributes to atmospheric pollution, which can seriously affect human health. Converting agricultural residues into biomass for biochar production is a relevant strategy for achieving the Sustainable Development Goals (SDGs).

The cultivation of date palms is widespread in arid and semi-arid regions worldwide, as they exhibit optimal growth in conditions characterized by extended periods of high temperatures, limited precipitation, and low humidity levels (Ahmad et al., 2012). Date palm tree farming covers one million hectares globally, with an estimated 105 million trees being grown. Date palm trees can amass many agricultural byproducts, such as desiccated leaves, fronds, trunks, seeds, and other similar materials. One date palm tree generates approximately 15 kg of biomass annually as agricultural waste, producing 600 million kilograms of green biomass. Globally, an estimated 50,000 tons of fronds from date palm trees are generated annually in regions where date palms are prevalent. According to Tahir, Al-Obaidy & Mohammed (2020), an estimated 20 kg of dry leaves are produced yearly. As a result, the annual production of date palm biomass is generated in large quantities. Date leftovers, including fronds, leaves, stems, rachis, and seeds, are abundant in nations that produce dates, such as Egypt and Saudi Arabia. These byproducts, however, are frequently dumped as waste.

Biochar is an organic amendment abundant in carbon and is produced by subjecting organic biomass to pyrolysis under high temperatures and the lack of oxygen. Biochar exhibits many benefits, including increasing soil carbon sequestration, mitigating GHG emissions, enhancing soil fertility, and expanding the soil's ability to retain moisture, thus enabling it to withstand periods of drought (Wang, Leng, Zhu, Wang, Gu & Yang, 2022). The *Phoenix dactylifera*, commonly known as the date palm, is a prevalent botanical species in North African countries such as Egypt and the Gulf region, notably Saudi Arabia. In date-producing countries, a significant quantity of waste is generated on farms, disposed of, or incinerated, contributing to environmental pollution. Disposing of waste in an environmentally sustainable manner poses a significant challenge, which can be addressed by utilizing biochar as an ideal solution. Biochar possesses distinctive control potential and, enhances soil health, sequesters soil carbon, rendering it a viable agricultural adaptation strategy with long-term benefits. The current understanding of biochar derived from date palm residue is limited, necessitating collaborative endeavours to inform the community engaged in date palm cultivation (Hansson, Haikola, Fridahl, Yanda, Mabhuye & Pauline, 2021).

Through the process of carbonization, the conversion of carbon into an aromatic structure occurs, resulting in a material that is comparatively more resistant to degradation than its raw counterpart. The proportion of gas, oil, and char is contingent upon the thermal conditions that are imposed upon the initial substance. The elemental composition of biochar, specifically regarding its carbon, potassium, and nitrogen content, directly correlates with the total biomass utilized, the duration of pyrolytic conditions, and the temperature at which said conditions are maintained. The physiochemical characteristics of biochar are influenced by various factors, including the constituents of the biochar and the conditions under which pyrolysis occurs, such as the temperature and duration of the process (Downie, Crosky & Munroe, 2009). Diverse forms of biomass exhibit differing configurations, including but not limited to essential components, moisture content, volatile content, and organic & inorganic content. Consequently, these variations impact the characteristics of the corresponding biochar, as Kloss et al. (2012) indicated.

The characteristics of biochar are significantly influenced by both the source of the feedstock and the specific conditions employed during the pyrolysis process. Generally, high-quality biochar is characterized by an elevated carbon (C) and minimal ash content (Mukherjee and Zimmerman, 2013, Singh et al., 2010). Several significant physicochemical features of good biochar include increased surface area and porosity, reduced bulk density, higher cation exchange capacity (CEC), and neutral to high pH levels (Mukherjee & Zimmerman, 2013). Additionally, it encompasses critical plant nutrients for crop growth and development, including nitrogen (N), phosphorus (P), and basic cations such as potassium (K), calcium (Ca), and magnesium (Mg) (Laghari et al., 2016).

Various agricultural residues are employed as biomass in the production of biochar. These include wheat and rice straw (Sun et al., 2019), rice husk (Armynah, Djafar, Piarah & Tahir, 2018), rape seed oil residues and palm oil (Bakar, Razak, Ahmad, Seh-Bardan, Tsong, & Meng, 2015), olive pomace (Ghouma, Jeguirim, Guizani, Ouederni & Limousy, 2017), and date seeds (Demirbas, 2017).

The thermal evaluation of date palm biomass, which included leaves, trunk bark, fronds, and seeds, was carried out in a stainless-steel container with an electrical muffle furnace (Lynch & Joseph, 2010). Different forms of biochar (leaves, branches, and stem barks) were obtained, and the pyrolysis temperature was kept between 300 and 600 °C for 4 h in the kiln under limited air supply at various charring overnight temperatures (Usman et al., 2015). Al-Wabel et al. (2019) created biochar from date palm leaflets by pyrolyzing a sufficient amount of biomass in a tube furnace at 600 °C for 3 h at a heating rate of 5 °C min^−1^ under limited oxygen circumstances. In addition, date palm fronds were carbonized in a muffle furnace at 400 °C for 30 min before being characterized using proximate analysis for application on soil properties (Khalifa & Yousef, 2015). A concentrated solar energy system for the pyrolysis of date palm waste to biochar is also constructed and simulated. This method provided long-term prospects for biochar production while lowering life cycle emissions and costs (Giwa, Yusuf, Ajumobi & Dzidzienyo, 2019). To the best of our current understanding, no prior research has been conducted to investigate the impact of continuous flow pyrolysis on the carbonization process of palm waste.

Therefore, this study aims to employ date palm frond (DPF) residues as biomass feedstock for producing high-quality biochar by utilizing a continuous screw-type pyrolysis furnace characterized by its ease of operation and maintenance.

## Material and methods

### Raw materials

**Fig. 1:**
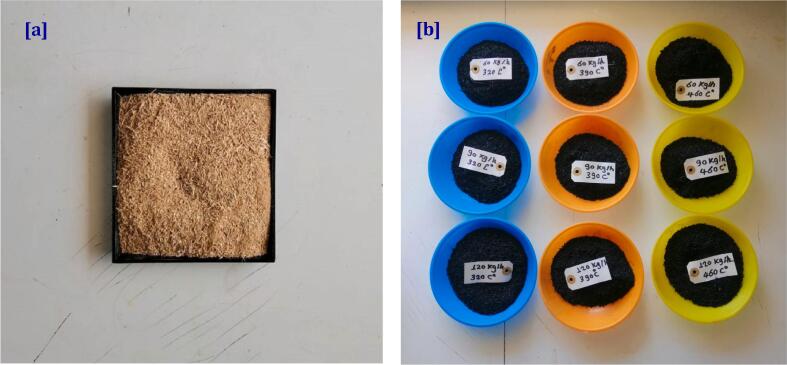
Biomass feedstock [a] and biochar produced [b] at different feeding rates and pyrolysis temperatures for date palm frond (DPF).

Date palm fronds (DPF) were employed as a feedstock material in the designed screw continues reactor to create biochar. The date palm fronds were supplied from the Central Lab of Date Palm Researches & Development, Giza, Egypt. The collected date palm fronds were air-dried and kept before being utilized in the studies. Table 1 shows the elemental & proximate DPF results with an EA 1112 elemental analyzer and a Perkin Elmer Thermo gravimetric analyzer. Fig. 1 displays a photo of DPF feedstock and biochar prior to performing elemental and proximate analysis.

**Table 1:** Characteristics and elemental & proximate analysis of used biomass materials (date palm fronds).

Elemental analysis, dry basis	Value
Carbon (%)	37.7 ± 1.31
Oxygen (%)	48.66 ± 1.96
Hydrogen (%)	5.58 ± 0.17
Nitrogen (%)	1.35 ± 0.10

Nutrient analysis, dry basis	
Sulfur (%)	0.53 ± 0.02
Ca, g/kg	0.73 ± 0.01
K, g/kg	1.58 ± 0.01
Mg, g/kg	0.09 ± 0.00
P, g/kg	0.05 ± 0.00
N, g/kg	1.36 ± 0.02

Proximate analysis, dry basis	
Moisture (%)	8.20 ± 0.72
Ash (dry) (%)	7.67 ± 1.05
Fixed carbon (%)	10.97 ± 1.00
Volatile compounds (%)	81.24 ± 2.64

Lignocellulosic composition, dry basis	
Cellulose (%)	33.80 ± 2.89
Hemicellulose (%)	28.01 ± 1.62
Lignin (%)	27.26 ± 0.46

### Structure and specifications of the developed biochar unit

**Fig. 2:**
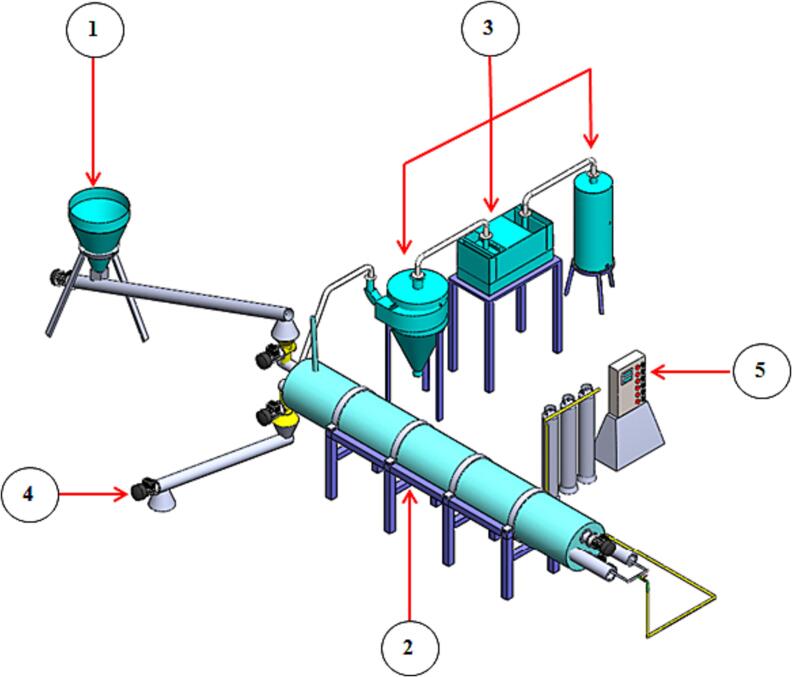
Isometric of the developed biochar unit. (1) Feeding unit, (2) Carbonization unit, (3) Filtration unit, (4) Discharge unit and (5) Control unit.

The biochar unit was designed, manufactured, and assessed by the Agricultural Engineering Research Institute (AEnRI), affiliated with the Agricultural Research Center (ARC), Egypt, in collaboration with the Academy of Scientific Research and Technology (ASRT), Egypt. The developed biochar unit consists of a feeding unit, carbonization unit, filtration unit, discharge unit and control unit, as shown in Fig. 2. Details and description of the developed biochar unit could be presented as follows:

#### The feeding unit

**Fig. 3:**
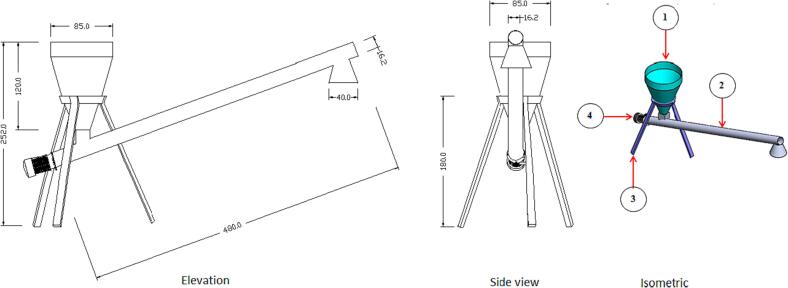
Views and isometric of the feeding unit. (1) Hopper, (2) Screw conveyor, (3) Subsidizing legs and (4) Electric motor.

The raw-material feeding unit consists of a conical shape hopper and an inclined screw conveyor for transferring the raw material to the carbonization reactor, as shown in Fig. 3. The hopper was made from an iron sheet with 0.3 cm thickness and 85 cm diameter. The hopper was rested over a frame with three subsidizing legs at a distance of 180 cm over the ground. The feeding materials travelled towards the carbonization unit through an inclined screw conveyor at an inclination angle of 14.5°, a length of 480 cm, a distance of 15.4 cm, and a screw pitch of 10.5 cm. The diameter of the screw housing was 16.2 cm, and a 1.5 kW electric motor was assigned to operate the screw conveyor.

#### The carbonization unit

**Fig. 4:**
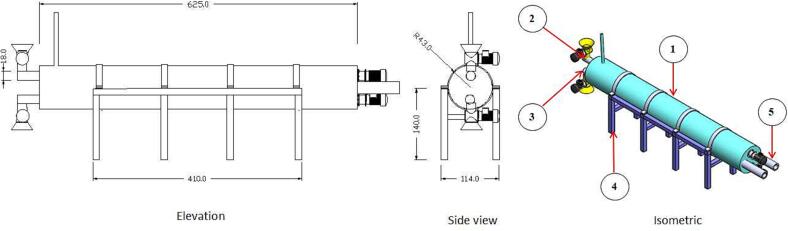
Views and isometric of the carbonization unit. (1) Carbonization-unit cylinder, (2) Forward screw, (3) Backward screw (4) Carbonization-unit chassis and (5) Heating-flam inlet.

The carbonization unit consists of a horizontal cylinder, two ways screw-conveyor, carbonization-unit chassis and heating-flam inlet, as shown in Fig. 4. The carbonization-unit cylinder is made of an iron sheet with 0.4 cm thickness, 86 cm diameter and 625 cm length. The two-way screw conveyor is fixed inside the cylinder and selected to reduce the total length of the carbonization unit. The forward and backward screw lengths are 520 and 475 cm, respectively. Each screw pitch and housing are 15.2 and 18 cm, respectively. An electrical motor of 2.2 kW was used to drive each screw. The carbonization unit was fixed over a chassis made of iron square with a 10 × 10 cm^2^ cross-section. The carbonization-unit chassis' overall length, width and height are 410, 114 and 140 cm, respectively. For heating the raw material, two heating-flam inlets are used. Two heating-flam inlets entered the flame on one side of the cylinder to heat the raw material.

#### The discharge biochar unit

The discharge biochar unit consists of a screw conveyor at an inclination angle of 14.5°, length of 465 cm, distance of 15.4 cm, and a screw pitch of 10.5 cm. The screw conveyor transfers the product biochar from the carbonization reactor to the outside. The diameter of the screw housing was 16.2 cm, and a 1 kW electric motor was assigned to operate the screw conveyor.

#### The filtration unit

**Fig. 5:**
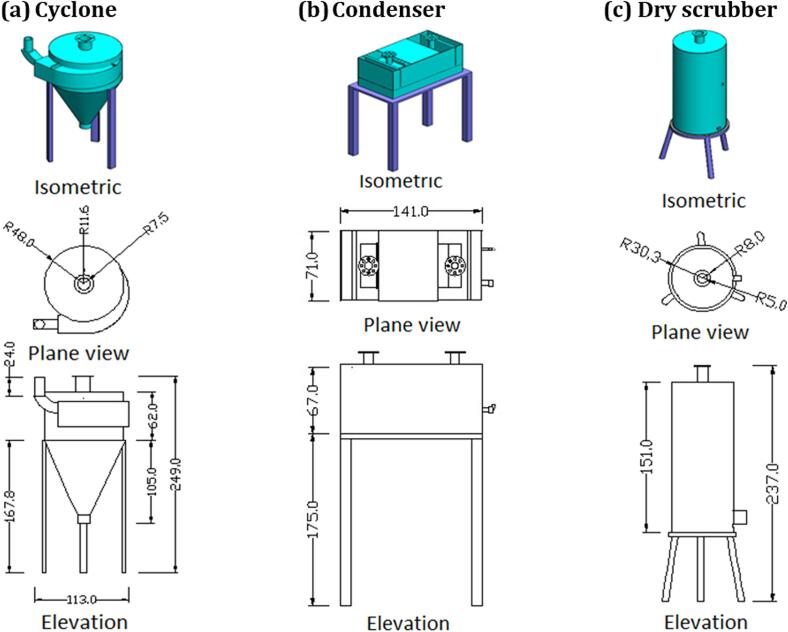
Isometrics and views of the filtration-unit components. Cyclone, (b) Condenser and (c) Dry scrubber.

The filtration unit consists of a cyclone, condenser, and dry scrubber, as shown in Fig. 5. The cyclone was made of iron sheet with 0.3 cm thickness. The cyclone consists of two welded parts (cylinder and conical shape part) mounted on three legs. The cylinder part has 96 cm diameter and 62 cm height. The conical shape part has 96 and 15 cm diameters and 105 cm height. The condenser's overall length, width and height are 141, 71 and 67 cm, respectively. The dry scrubber was made of iron sheet with 0.3 cm thickness, 60.6 cm diameter and 151 cm height. The units treat the smoke and separate it into different components: coal tar exits from the cyclone, and bio-oil exits from the dry scrubber. The condenser with a water cooler condensed the gas by cooling it to produce distilled compounds (bio-oil). A suction fan was assigned to suck the produced syngas from the syngas tank and re-circulate it through the heating system to reduce heating gas consumption for operating and controlling the unit parts; a control unit was assigned. The unit included switches for motors, an inverter for changing the speed of feeding motors, and indicators for the temperature sensors.

### Experimental site & test procedure

The experimental trials were conducted at the Rice Mechanization Center (RMC), Kafr El-Sheikh Governorate, Egypt. The raw material for the experimental work was taken from the date palm fronds (DPF). The fronds were chipped to a length of 2 cm using a double knife drum chopper (TOMCAT Model 200 AFE, South Africa) and then ground in fine particles. The moisture content of the chipped fronds was 8.20 %w.b.

### Methods

The carbonization unit was operated using a Liquid fuel Petroleum gas (LPG) with a calorific value of 431 MJ kg^−1^. The gas supply was used for the ignition process, and the full energy supply at the beginning of the operation process as the unit approached the required level of heating temperature for the pyrolysis process (320–460 °C) and reached a stabilized operation condition (two working hours are required). The chipped date palm fronds were fed to the feeding hopper, and the produced syngas were recycled to the heating system, which shared the total required energy.

The experimental work proceeded after reaching unit stabilization. The experimental work proceeded after getting unit stabilization. Three different heating temperature levels (320, 390, and 460 °C) and three different feeding rates (60, 90, and 120 kg/h) were examined for biochar production. Samples from produced biochar were taken at the end of each experimental run, and the following analysis proceeded.

#### Scanning electron microscopy (SEM)

The morphological changes in the biochar samples were evaluated through scanning electron microscopy (SEM, Hitachi, S-570) under high vacuum conditions, with an accelerating voltage of 20 kV and a magnification of 12000x, following the methodology described by Joardder, Halder, Rahim & Masud (2017).

#### Fourier transforms infrared spectroscopy (FT-IR) analysis

The attenuated total reflectance (ATR) method was utilized to assign Fourier transforms infrared spectroscopy (FT-IR) in the 600–4000 cm^−1^ range (El Sharkawi, Tojo, Chosa, Malhat, & Youssef, 2018). In addition, 0.5 mg from each sample were deposited onto the Ge window of a Nicolet FT-IR instrument equipped with an attenuated total reflectance accessory after being comminuted to a particle dimension of 0.1 mm. The spectra of the samples were subjected to analysis through the employment of a KBr beam sampler, which involved over 256 scans. The Fourier Transform Infrared (FT-IR) data was obtained utilizing a diamond Attenuated Total Reflectance (ATR) accessory, with a spectral resolution of 4 cm^−1^ and an average of 32 scans.

#### Surface area and pore properties

The surface area and total volume of DPF biochar were determined using the Brunauer-Emmet Teller (BET) method, which involves measuring nitrogen adsorption–desorption isotherms at a temperature of −196 °C (Hung, Tsai, Chen, Lin, & Chang, 2017). This was carried out using an accelerated surface area and porosimetry system manufactured by Quantochrome Instruments in the United States. BET surface area was determined by analyzing the adsorption data within the pressure range of 0.05 to 0.25, and the resulting value was expressed in units of m^2^g^−1^. Determining the total pore volume involved the conversion of the measured quantity of nitrogen gas adsorbed, expressed in cm^3^g^−1^ at standard temperature and pressure, at a relative pressure of 0.98, into the volume occupied by the adsorbate in its liquid form.

The determination of water holding capacity involved the computation of the quantity of water, expressed in g /g water, that was retained by a 10 g biochar specimen on a dry weight basis (d.b). The biochar specimens were subjected to a drying process at a temperature of 105 °C for 15 h. Subsequently, the dried samples were inserted into funnels equipped with filter paper. Deionized water was introduced into the funnels constantly over 15 min. Following the immersion of the biochar samples in water, any surplus water was eliminated through the lower section of the funnel (Saarnio, Heimonen & Kettunen, 2013).

#### pH, electric conductivity (EC), and cation exchange capacity (CEC)

The pH of biochars was determined using the method established by Joardder, Halder, Rahim & Masud (2017). A solution of biochar was prepared by dissolving 4.0 g of the substance in water using a 100 mL conical flask. The flask was filled with boiling water and covered with a watch glass. The solution was then permitted to cool, following which the supernatant was drained. Following the completion of the process, the liquid portion was allowed to reach room temperature and subsequently subjected to pH measurement utilizing a pH meter (Metrohm 827 pH Lab, USA). The biochar's electrical conductivity (EC) was determined using an EC meter (CON 700 model-Eutech Instruments, USA). The biochar samples were moistened with deionized water using a solid-to-water ratio of 1:5. The mixture was then stirred for 24 h prior to conducting electrical conductivity (EC) measurements.

Cation exchange capacity (CEC) was measured using the technique described by Song & Guo (2012). A total of 0.50 g of biochar was mixed with 40 ml of a 1 M solution of ammonium acetate in a 50-millilitre Falcon tube. After that, the mixture was thoroughly mixed for a full 24 h. After filtering the liquid with a vacuum pump, 40 mL of ammonium acetate was added. The vacuum pump was then decanted with 30 mL of isopropanol in three equal portions. The leftover biochar was leached with four 50-mL doses of 1 M KCl, and the leachate was collected. An auto-analyzer (QuikChem 8000 Series FIA + System; Lachat Instruments, USA) was used to assess how much NH4 + was extracted, and atomic absorption spectrometry (AAnalyst 400; PerkinElmer, USA) was used to determine how many exchangeable cations were found in the biochar.

#### Proximate & elemental composition analysis

The biochar yield was determined in Eq. (1), as indicated by Lynch & Joseph (2010):

(1) $$\mathrm{Biochar}\;yield\;\left(\%\right)=\frac{M_{1}}{M_{2}}\times 100\;\;\;\;\;\;\;\;\;\;\;\;\;\;\;\;\;\;\;\;\;\;\;\;\;\;\;\;\;\;\;\;\;\;\;\;\;\;\;\;\;\;\;\;\;\;\;\;\;\;\;\;\;\;\;\;\;\;\;\;\;\;\;\;\;\;\;\;\;\;\;$$

where:

$M_{1}$ is the biochar mass (g).

$M_{2}$ is the dried raw material mass by air oven (g).

The material's moisture content was determined using the indirect drying method provided in the EN ISO 18134–3:2015–11 standard (Łapczyńska-Kordon, Ślipek, Słomka-Polonis, Styks, Hebda & Francik, 2022). A 1 g sample of the material under study was dried in a laboratory oven with forced air circulation and thermostatic temperature control. For 90 min, the drying procedure was carried out at 110 °C. Following the completion of the drying process, the samples were transported to a desiccator and weighed on a digital laboratory scale with an accuracy of 0.0001 g. Eq. (2) was used to calculate the moisture content (MC) as follows:

(2) $$Moisture\;content\;\left(MC\right)\;=\frac{M_{W}-M_{D}}{M_{W}}\times 100\;\;\;\;\;\;\;\;\;\;\;\;\;\;\;\;\;\;\;\;\;\;\;\;\;\;\;\;\;\;\;\;\;\;\;\;\;\;\;\;\;\;\;\;\;\;\;\;\;\;\;\;\;\;\;\;$$

where:

$M_{W}$ is the initial biochar mass (g)

$M_{2}$ is biochar mass after drying (g)

The dry combustion technique was employed to determine the ash content. In summary, 5.0 g of biochar was thermally treated at 500 °C for 8 h. After the crucible had reached room temperature, it was re-weighed, as stated by Joardder, Halder, Rahim & Masud (2017). Ultimately, the ash percentage was calculated through the utilization of a formula (Eq. (3):

(3) $$\mathrm{Ash}\;content\;\left(\%\right)=\frac{M_{\mathrm{Ash}}}{M_{1}}\times 100\;\;\;\;\;\;\;\;\;\;\;\;\;\;\;\;\;\;\;\;\;\;\;\;\;\;\;\;\;\;\;\;\;\;\;\;\;\;\;\;\;\;\;\;\;\;\;\;\;\;\;\;\;\;\;\;\;\;\;\;\;\;\;\;\;\;\;$$

where:

$M_{\mathrm{Ash}}$ is the ash mass (g)

The American Society for Testing and Materials (ASTM) D5142 method (ASTM, 2009) was employed to ascertain the levels of volatile matter (VM) present in the sample. The volatile matter (VM) content was ascertained by heating the char in a covered crucible and maintaining it at a temperature of 950 °C for 7 min. This procedure aimed to evaluate the impact of VM on weight loss. The calculation of fixed C content was performed using the following Eq. (4) (Venkatesh et al., 2022):

(4) $$\mathrm{Fixed}\;carbon\;\%=100\%-\left(Ash\;\%+Volatile\;matter\;\%\;\right)\;\;\;\;\;\;\;\;\;\;\;\;\;\;\;\;$$

The carbon (C), hydrogen (H), oxygen (O), and nitrogen (N) composition of biochar was determined using an X-ray fluorescence spectrometer (XRF) produced by Malvern Panalytical Almelo, the Netherlands (CNHOS). The quantity of phosphorus (P) was determined using the Bray II methodology (Song & Guo, 2012). The analysis of tradable elements, namely potassium (K), magnesium (Mg), aluminium (Al), and silicon (Si), was conducted using the method proposed by Samsuri, Sadegh-Zadeh & Seh-Bardan (2014).

### Statistical analysis

The data were analyzed using the SPSS 26.0 software (Version 26, IBM Corporation, USA). A two-way analysis of variance (ANOVA) was utilized to conduct multiple comparisons, followed by a post hoc analysis using the Tukey test. Statistical significance was determined at the 0.05 level. The biochar characterization parameters were subjected to Principal Component Analysis (PCA) using Minitab Pro v.21.2, and a heatmap was generated to perform correlation analysis using Origin 2021 software (Origin Lab, Northampton, MA, USA).

## Results and discussions

### Scanning electron microscopy (SEM)

**Fig. 6:**
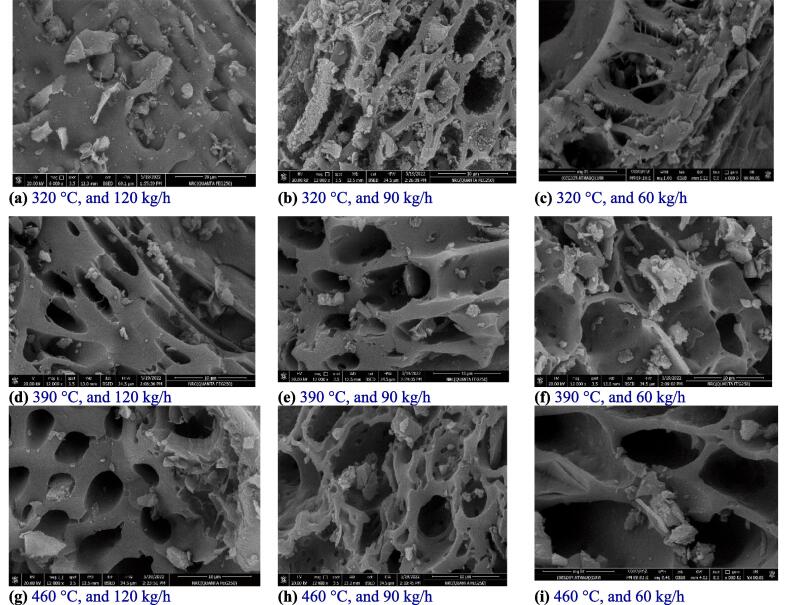
SEM images were captured at 320, 390, and 460 °C pyrolysis temperatures. Each row was fed 60, 90, and 120 kg/h from right to left.

The morphology of biochar was assessed through the utilization of SEM analysis. Fig. 6[a-i] illustrates the effects of different pyrolysis temperatures and feeding rates on biochar, as observed through scanning electron microscopy (SEM) analyses. The biochar samples exhibited porous surfaces due to the volatilization of organic materials. Significant channels and pores within biochar were manifested as the pyrolysis temperature increased and the feeding rate decreased. Upon exposure to a temperature of 320 °C, the surface of palm fronds exhibited a smooth and uniform texture devoid of internal pores, as depicted in Fig. 6[a-c]. This observation suggests the existence of cellulose, hemicellulose, and lignin, which transform a porous and amorphous state following the application of heat treatment. However, at 460 °C, the biochar's morphology changed to that of a honeycomb, with cylindrical holes interrelated by some large holes. As Guo & Lua (1998) suggested, Biochar with well-organized pore structures has a large BET and adsorptive capacity. Surface cracks and shrinkage were seen after heating the biochar to 460 °C at various feeding rates. The particles in 460 °C pyrolyzed biochar are extremely porous, hollow, spherical, and well-organized (Fig. 6 [c, f, and i]). The buildings' thin walls gave them a frail aspect. When the pyrolysis temperature was elevated, the structure of biochar became more organized, with a drop in the number of micropores and an increase in the number of big pores.

### Fourier transforms infrared spectroscopy (FT-IR) analysis

**Fig. 7:**
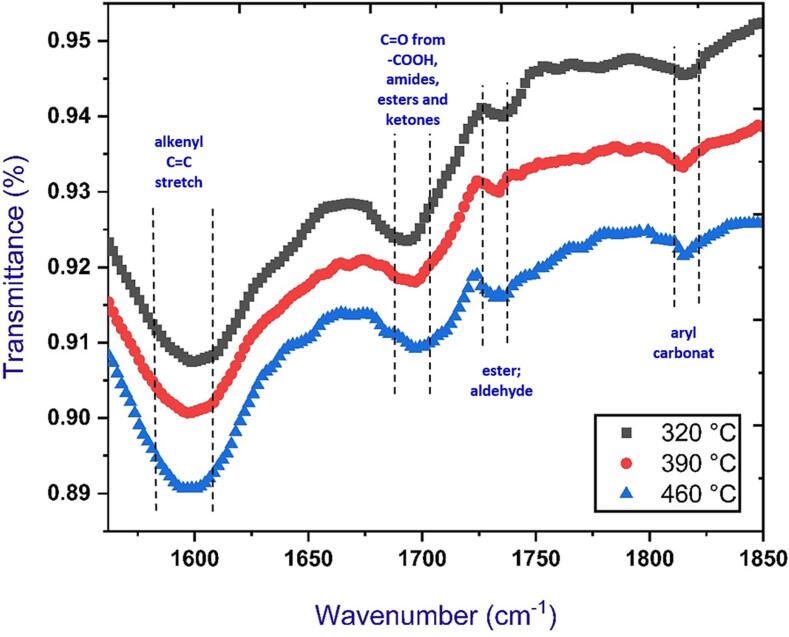
FTIR spectra of biochar produced from date palm fronds as a function of wavenumber at various studied pyrolysis temperatures.

Fig. 7 displays the Fourier-transform infrared spectra of biochar obtained from date palm fronds that underwent pyrolysis at temperatures of 320, 390, and 460 °C. Although FTIR was successfully utilized to examine the influence of temperature on biochar, the results of the feeding rates impact were removed because there was no visible variation in intensity with the different feeding rates. The spectral feature observed at a wavenumber of 1596 cm^−1^ corresponds to the stretching vibrations of alkenyl C=C bonds. The observed phenomenon may also communicate to the H—O—H bending band of water, which exhibits a decreasing trend as the pyrolysis temperature is raised. The spectral band followed at a wavenumber of 1695 cm^−1^ indicates the presence of C=O functional groups, which may arise from various chemical species such as –COOH, ketones, amides, and esters (Zojaji, Esfandiarian & Taheri-Shakib, 2021). The spectral band observed at a wavenumber of 1731 cm^−1^ indicates the presence of esters and aldehydes. The band located at 1814 cm^−1^ indicates aryl carbonate, as indicated in (Sizirici, Fseha, Yildiz, Delclos & Khaleel, 2021). Tomczyk, Sokołowska & Boguta (2020) reported that biochar produced through pyrolysis at low temperatures exhibits functional properties comparable to its feedstock. The utilization of FT-IR analysis revealed that biochar underwent chemical alterations throughout the pyrolysis process at different temperature intervals. The dehydration of frond feedstocks occurred as the pyrolysis temperature was elevated, converting aliphatic bonds to aromatic bonds that ultimately formed stable graphene (Zama, Zhu, Reid & Sun, 2017). The sorption properties of frond biochar samples are attributed to the presence of carboxyl, hydroxyl, and amino groups. As illustrated in Fig. 7, these groups' concentration declines as the pyrolysis temperature increases. The frond biochar samples may be influenced by diverse functional groups, including those that contain oxygen functional groups. These groups can potentially affect the surface reactions, hydrophilicity, and electrical and catalytic characteristics of the samples, as indicated by a previous study (Yang et al., 2019).

### Proximate, elemental, and nutrient composition analysis

The biochar yields significantly declined from 50.07 to 35.13 % as the pyrolysis temperature was elevated from 320 to 390 ◦C at the 120 kg/h feeding rate. In addition, the same range of increasing the pyrolysis temperature for the feeding rate of 60 kg/h reduced biochar yield from 48.17 to 31.70 %.The probable cause for this occurrence is attributed to the decomposition of a significant portion of the lignocellulosic material within this particular temperature range, as indicated by Intani, Latif, Kabir & Müller (2016). Upon increasing the pyrolysis temperature from 390 to 460 ◦C, the biochar yield decreased from 48.17 to 25.90 % and from 48.90 to 26.57 % at feeding rates of 60 and 90 kg/h, respectively, as displayed in Table 2. The outcome of this analysis suggests that a significant portion of the volatile matter has been extracted (Zhao, Ta & Wang, 2017).

**Table 2:** Proximate & elemental analysis of date palm fronds (DPF) biochar under each treatment.

Biochar characteristics	Pyrolysis Temperature, °C	320 °C			390 °C			460 °C		
	**Feeding rate, kg/h**	**60**	**90**	**120**	**60**	**90**	**120**	**60**	**90**	**120**
Proximate analysis, (%) dry basis	**biochar yield (%)**	48.17 ± 0.47b	48.90 ± 0.36b	50.07 ± 0.25 a	31.70 ± 0.44 d	34.07 ± 0.40c	35.13 ± 0.42c	25.90 ± 0.30f	26.57 ± 0.31 ef	28.20 ± 0.61 e
	**Moisture content (%)**	7.95 ± 0.03 bc	8.04 ± 0.05b	8.17 ± 0.02 a	7.22 ± 0.00 e	7.41 ± 0.04 d	7.93 ± 0.06c	5.66 ± 0.04h	6.00 ± 0.03 g	6.30 ± 0.04f
	**Ash content (%)**	6.80 ± 0.44 de	5.53 ± 0.50 e	5.17 ± 0.38 e	10.85 ± 0.13 ab	9.42 ± 0.37c	7.69 ± 0.45 d	11.73 ± 0.38 a	10.67 ± 0.21b	10.30 ± 0.30 bc
	**Volatile matter (%)**	43.40 ± 0.53 a	42.20 ± 0.26 a	43.53 ± 0.35 a	22.17 ± 0.86c	23.91 ± 0.31c	27.63 ± 1.21b	10.66 ± 0.26 e	12.13 ± 0.15 e	15.13 ± 1.27 d
	**Fixed carbon (%)**	49.80 ± 0.90f	52.27 ± 0.71 e	51.30 ± 0.53 ef	66.98 ± 0.78c	66.67 ± 0.67cd	64.68 ± 0.93 d	77.61 ± 0.50 a	77.20 ± 0.07 a	74.57 ± 1.40b
Elemental analysis, (%) dry basis	**Carbon (%)**	72.03 ± 1.11 d	69.68 ± 0.48 de	67.97 ± 0.80 e	80.92 ± 0.83b	79.60 ± 0.79b	77.03 ± 1.10c	83.60 ± 0.30 a	82.40 ± 0.82 ab	82.37 ± 1.07 ab
	**Hydrogen (%)**	4.90 ± 0.04b	5.35 ± 0.04 ab	5.93 ± 0.03 a	3.01 ± 0.03c	3.20 ± 0.05c	3.38 ± 0.02c	1.96 ± 0.05 d	2.05 ± 0.04 d	2.15 ± 0.05 d
	**Oxygen (%)**	15.06 ± 1.20b	18.60 ± 0.96 a	21.09 ± 0.66 a	5.00 ± 0.75 de	7.27 ± 0.61 d	11.26 ± 1.27c	2.62 ± 0.11 e	4.64 ± 0.97 e	4.67 ± 0.80 e
	**Nitrogen (%)**	0.18 ± 0.01 d	0.84 ± 0.02 a	0.88 ± 0.06 a	0.22 ± 0.02 d	0.51 ± 0.03c	0.63 ± 0.03b	0.09 ± 0.01 e	0.24 ± 0.01 d	0.51 ± 0.02c
Nutrient analysis, (%) dry basis	**P, g/kg**	0.102 ± 0.008 d	0.093 ± 0.006 d	0.077 ± 0.006 d	0.133 ± 0.006cd	0.110 ± 0.010cd	0.100 ± 0.010 d	0.447 ± 0.035 a	0.333 ± 0.029b	0.150 ± 0.010c
	**K, g/kg**	2.187 ± 0.055	2.243 ± 0.051	2.107 ± 0.168	2.627 ± 0.061	2.570 ± 0.066	2.473 ± 0.025	2.853 ± 0.035	2.787 ± 0.091	2.717 ± 0.031
	**Ca, g/kg**	5.483 ± 0.111	5.443 ± 0.055	5.293 ± 0.070	6.897 ± 0.184	6.570 ± 0.524	6.477 ± 0.178	8.493 ± 0.122	8.051 ± 0.069	7.750 ± 0.207
	**Mg, g/kg**	1.420 ± 0.010f	1.403 ± 0.015f	1.317 ± 0.015f	1.843 ± 0.040c	1.703 ± 0.006 d	1.587 ± 0.025 e	2.347 ± 0.023 a	2.203 ± 0.074b	2.137 ± 0.055b

* The mean ± standard deviation (SD) was used to express the data; means followed by distinctive lowercase letters indicate significantly different results (P<0.05) at a 5% significance level.

* The absence of letters indicates the insignificance of differences between treatments.

The moisture content of the biomass was determined to be 8.20 % on a wet basis (w.b). In the case of the produced DPF, the moisture content varied between 5.66 % and 8.17 % w.b. The lowest value was observed in the biochar sample tested under the operating conditions of 460 °C and 60 kg/h, as indicated in Table 1, Table 2. The moisture content of biochar produced under different feeding and temperatures varied significantly (P < 0.05, HSD_0.05_ = 0.105). Similar results were found from the effect of the carbonization process on goldenrod plants (Łapczyńska-Kordon, Ślipek, Słomka-Polonis, Styks, Hebda & Francik, 2022). Following the carbonization process, the moisture content of biochar is reduced due to the evaporation of water during the thermal treatment of biomass. The disparities in the moisture content between the fresh biomass and biochar significantly influence the calorific value (Alba-Reyes, Perez-Gil, Barrera, Casas-Ledon & Arteaga-Perez, 2022).

The study found that the biochar produced had a variable content of volatile matter (ranging from 10.66 % to 43.53 %) and fixed carbon (ranging from 49.80 % to 77.61 %). Elevating the pyrolysis temperature resulted in a noteworthy reduction in the volatile matter (VM) content; conversely, an inverse pattern was observed for the fixed carbon. No significant variations in the fixed carbon content and VM were observed at different feeding rates under identical temperature conditions (Table 2). This phenomenon could be attributed to the elevated temperature, which caused the volatile matter to break down into lower molecular weight liquids and gases rather than biochar formation (Ronsse, Van Hecke, Dickinson & Prins, 2013). Simultaneously, the temperature rise may lead to the dehydration of hydroxyl groups and the thermal degradation of lignin and cellulose, as stated by Zhang et al. (2015). The findings of this study validate that the temperature rise positively impacts biochar's stability through the depletion of volatile components (Zornoza, Moreno-Barriga, Acosta, Muñoz & Faz, 2016).

The increased ash content from 5.17 to 10.85 % was significant as the pyrolysis temperature was raised from 320 to 390 °C and a decreased feeding rate from 120 to 60 kg/h. The augmentation in ash content was due to a gradual accumulation of inorganic components (Southavong et al., 2018), a finding substantiated by our nutrient evaluation (Table 2). With a rise in pyrolysis temperature from 390 to 460 °C, it is possible for certain inorganic materials to undergo volatilization in the form of gas or liquid. As a result, the ash content remained stable at a higher temperature (460 °C).

The elemental composition of DPF biochar is presented in Table 2. By ANOVA analysis, the carbon percentage differed significantly throughout exposure for different pyrolysis temperatures at various feeding rates (p < 0:05; HSD_0.05_ = 2.437). At 60 and 120 kg/h feeding rates, the carbon content (C) ranged (72.03–83.60 %) and (67.97–82.37 %), respectively. The highest attainable percentage, amounting to 83.60 %, was obtained when the operating conditions were set at a feeding rate of 60 kg/h and a temperature of 460 °C. An increase in pyrolysis temperatures has been observed to lead to higher carbon content in biochar, indicating that carbonation is enhanced at higher pyrolysis temperatures (Sizirici, Fseha, Yildiz, Delclos & Khaleel, 2021). The observed decrease can be attributed to the elevated degree of polymerization of biochar, leading to a denser carbon structure (Tomczyk, Sokołowska & Boguta, 2020). The escalation in carbon levels could potentially account for the depletion of hydrogen and oxygen constituents from the biochar as the temperature of pyrolysis increases, as posited by Antal & Grønli (2003). At the same time, the oxygen and hydrogen levels significantly decreased (P < 0.05, HSD_0.05_ = 2.508 and 0.591, respectively) as the carbonization temperature increased. The observed declines can be attributed to the dehydration-induced removal of surface functional groups containing O and H during pyrolysis (Z. Zhang et al., 2015). As the corresponding pyrolysis temperature increased, the total nitrogen content significantly decreased (P < 0.05, HSD_0.05_ = 0.083) from 0.18 to 0.09 and 0.88 to 0.51 at the lowest and maximum feeding rates, respectively (Table 2). The conversion of nitrogen-containing structures such as amino sugars, amino acids, and amines into heterocyclic N aromatic structures occurs during the pyrolysis of plant biomass (Cao & Harris, 2010). This implies that the nitrogen will remain accessible and will not undergo immediate decomposition but will instead be released over an extended carbonation period.

Table 2 displays the element components of P, K, Ca, and Mg for the produced DPF biochar. There were clear significant effects of pyrolysis temperature and feeding rate on P and Mg (P < 0.05, HSD_0.05_ = 0.048 and 0.104, respectively) and no significant impact on K and Ca (P > 0.05), where the maximum values were observed at 460 °C and 60 kg/h. Based on the X-ray fluorescence spectroscopy (XRF), Ca, K, and Mg is the most abundant ash element in DPF biomass with percentages of 8.493, 2.853, and 2.347 g/kg at 60 kg/h feeding rate and 460 °C temperature, respectively.

### Surface area and pore properties

The BET surface areas of the produced biochar raised with the increase of the pyrolysis temperature at the studied range of (320–450 °C), as shown in Table 3. At 320, 390, and 460 °C, the BET surface areas for 60 kg/h feeding rate were 227.33, 338.00, and 361.00 m^2^/g, respectively, and for the rate of 120 kg/h, the corresponding BET surface areas were 180.33, 270.67, and 304.67 m^2^/g, respectively. The corresponding values were greater at 460 °C, possibly because of the vigorous reactions at this temperature, which resulted in the formation of biochar with mesoporous pores. The volatile material evaporates as the temperature rises, creating new pores and providing supplementary surface area (Sizirici, Fseha, Yildiz, Delclos & Khaleel, 2021).

**Table 3:** Brunauer Emmet-Teller (BET) surface area, total pore volume, and water holding capacity of DPF biochar under different pyrolysis temperatures (320, 390, and 460 °C) and feeding rates (60, 90, and 120 kg/h).

Pyrolysis Temperature, °C	Feeding rate, kg/h	Physical properties		
		BET surface area (m^2^/g)	Total pore volume (cm^3^ g^−1^)	Water holding capacity (g_water_/10 g)
320	60	227.33 ± 4.60 e	0.205 ± 0.008f	6.04 ± 0.04 de
	90	198.00 ± 2.00f	0.178 ± 0.004 g	6.16 ± 0.13 d
	120	180.33 ± 5.51 g	0.148 ± 0.007 h	5.94 ± 0.04 e
390	60	338.00 ± 2.00b	0.299 ± 0.002c	6.87 ± 0.02 a
	90	306.00 ± 5.29c	0.249 ± 0.002 d	6.61 ± 6.36b
	120	270.67 ± 3.06 d	0.221 ± 0.005 e	6.36 ± 0.02c
460	60	361.00 ± 6.56 a	0.336 ± 0.004 a	6.17 ± 0.06 d
	90	329.33 ± 3.79b	0.318 ± 0.002b	6.20 ± 0.02 d
	120	304.67 ± 5.03c	0.304 ± 0.003c	6.28 ± 0.02 cd

* The mean ± standard deviation (SD) was used to express the data; means followed by distinctive lowercase letters indicate significantly different results (P < 0.05) at a 5 % significance level.

The total pore volume of pyrolyzed DPF biochar has significantly increased (P < 0.05, HSD_0.05_ = 0.013) with increasing temperature and decreasing feeding rate, as presented in Table 3. Extended pyrolysis at > 700 °C expands the pores, causing structural contraction and size reduction (Liu, Fang & Wang, 2008). Thus, the carbonization process in this study was carried out in a suitable temperature range (320–460 °C) to increase the total pore volume.

The hydrophilic properties of biochar are crucial for promoting optimal plant growth, particularly in soil types with low water holding capacity, such as sandy soil. Table 3 revealed that with increasing temperatures and decreasing feed rates up to (390 °C and 60 kg/h), the water holding capacity of DPF biochar was progressively increased (p < 0.05, HSD_0.05_ = 0.158) in the range 5.94–6.87 g_water_/10 g. Then, the water holding capacity decreased by increasing the temperature to 460 °C. Kong, Loh, Bachmann, Zainal & Cheong (2019) achieved a similar trend in producing palm kernel shell (PKS) biochar. At the beginning of the heat processing, the biochar pores were softened and became more exposed. However, when the pores became larger, at operation conditions of (460 °C and 60 kg/h), they could not hold the water as effectively as smaller pores. This DPF biochar retained less water in the higher heat treatment condition (460 °C) when the total pore volume reached 0.336 cm ^3^ g^−1^.

### pH, electric conductivity (EC), and cation exchange capacity (CEC)

**Fig. 8:**
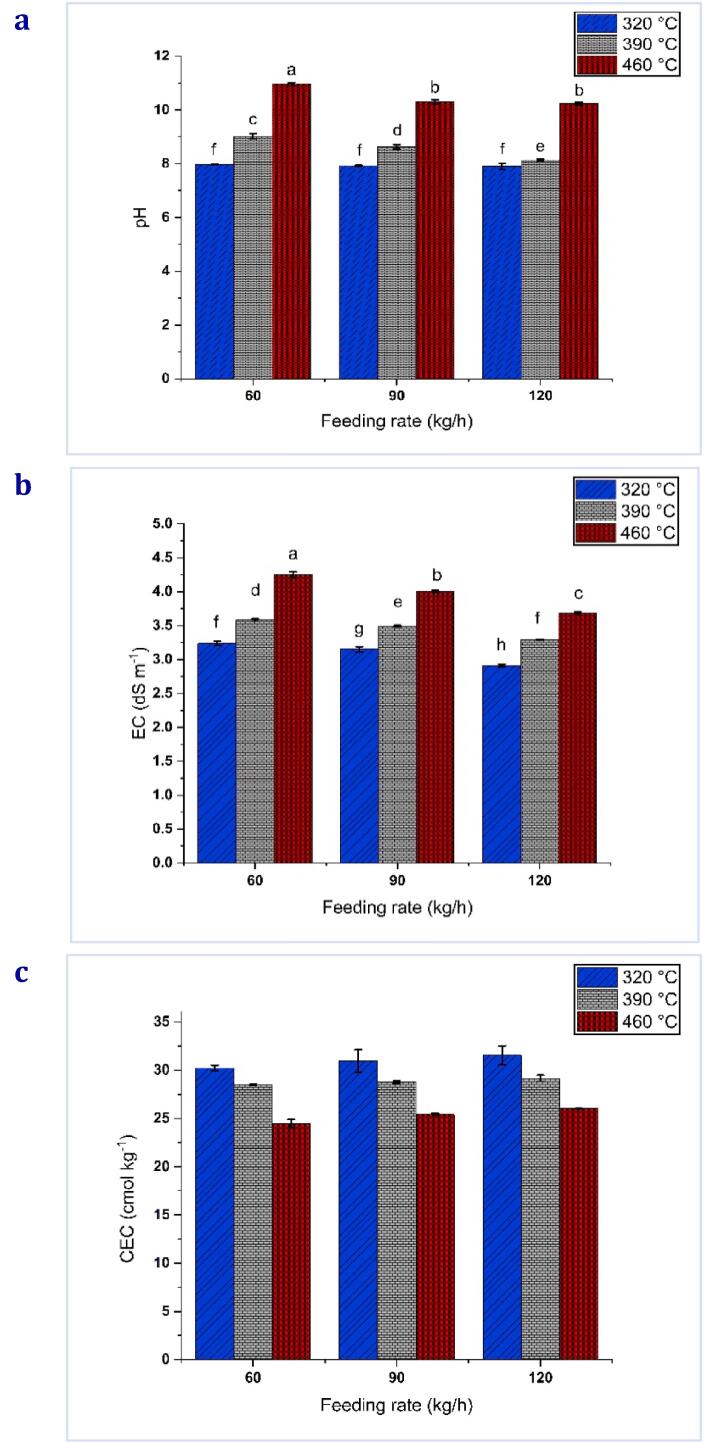
Effect of the pyrolysis temperatures (320, 390, and 460 °C) and feeding rate (60, 90, and 120 kg/h) on [a] pH, [b] EC, and [c] CEC. * The mean ± standard deviation (SD) was used to express the data; means followed by distinctive lowercase letters indicate significantly different results (P < 0.05) at a 5 % significance level. The absence of letters indicates the insignificance of differences between treatments.

pH of biochar was increased significantly (p < 0.05, HSD_0.05_ = 0.172) with the rise of pyrolysis temperature and down feed rate, as presented in Fig. 8[a]. The produced biochars exhibited an alkaline nature, as evidenced by their pH values ranging from 7.96 to 10.96 at a 60 kg/h feeding rate and from 7.90 to 10.24 at 120 kg/h, which have consistently reported that biochars tend to have an alkaline nature (Ahmad et al., 2012, Zhang et al., 2015). The pH of biochar produced at 320 °C exhibited a lower value than biochar produced at temperatures ranging from 390 to 460 °C. This trend was particularly evident as the pyrolysis temperature increased. The observed increments in charring temperature can be attributed primarily to the process of liming, which results in the reduction of acidic functional groups and the concomitant augmentation of basic functional groups. Separating alkali salts from organic compounds also contributes to this phenomenon (Southavong et al., 2018). The findings of this study indicate that the pH of DPF biochar is more significantly influenced by the pyrolysis temperature than the feeding rate. The study by Mukherjee & Lal (2014) also found that a significant portion of the biochar used for soil amendment is alkaline biochar, as produced in this study. During the carbonation process, acidic functional groups are removed, while the concentration of salts derived from alkali and alkaline soil elements increases (Fuertes et al., 2010). These salts include four classes: (1) easily soluble salts, (2) carbonates, (3) moderately soluble metal oxides and hydroxides, and (4) silicates. Most of these salts are provided by biochar, which is alkaline and important for soil (Wang et al., 2014).

Fig. 8[b] displays the biochars' electrical conductivity (EC) values produced from diverse feeding rates under different pyrolytic temperatures ranging from 320 to 460 °C. All biochar samples' electrical conductivity (EC) values exhibited a 2.91 to 4.25 dSm^−1^ range across various operational conditions. The feed rate and pyrolytic temperature significantly affected the produced biochars' EC (P > 0.05, HSD_0.05_ = 0.076). The (EC) values generally exhibited an upward trend as the pyrolytic temperature increased and the feeding rate decreased across all biochars. The biochar DPF generated under the conditions of 460 °C and 60 kg/h demonstrated the greatest (EC) values (4.25 dSm^−1^) relative to the other biochars, which can be attributed to the increased presence of soluble salts (Singh et al., 2021). An increase in temperature and a decrease in feed rate resulted in a corresponding increase in ash content, consistent with the observed trend of elevated electrical conductivity. The concentration of components within the ash content can be attributed to the loss of volatiles (Cantrell, Hunt, Uchimiya, Novak & Ro, 2012), which caused the elements to become more localized. This phenomenon is attributed to the enhanced mobility of the K + ion within the biochar matrix, likely due to the higher proportion of mineral ash and its consequent increased electrical conductivity (Panahi et al., 2020). Applying biochar (with appropriate electrical conductivity) significantly increases the electrical conductivity of the soil. The rise in nutrient levels can be attributed to the release of unbound nutrients (cations and anions) from the biochar, which leads to their presence in the soil solution and their subsequent availability for plant uptake (Tag, Duman, Ucar & Yanik, 2016). However, applying biochar with high EC values ​​must be prohibited as it can reduce seed germination and crop productivity with higher sensitivity to salt in the soil solution (Qian et al., 2023).

Biochar's cation exchange capacity (CEC) is a significant property that denotes its ability to adsorb cationic nutrients (Tag, Duman, Ucar & Yanik, 2016). The findings indicate that the cation exchange capacity (CEC) of the produced biochars non-significant decrease (P > 0.05), ranging from 31.54 to 24.47 cmol.kg^−1^, despite an increase in pyrolysis temperature from 320 to 460 °C and a decrease in feeding rate from 120 to 60 kg/h, as Fig. 8[c] presents. A rise in the temperature during the pyrolysis process results in a decrease in CEC. This reduction can be attributed to the oxidation of aromatic carbon and the generation of carboxyl groups in biochar (Zornoza, Moreno-Barriga, Acosta, Muñoz & Faz, 2016). The impact of phenolic, hydroxyl, quinone, and carbonyl groups on biochar's cation exchange capacity (CEC) is significant. The Fourier Transform Infrared (FTIR) spectra, as depicted in Fig. 7, indicate a decrease in unbound hydroxyl (–OH) group concentration with the rise in pyrolysis temperatures.

### Principal component analysis (PCA) and correlation study

**Fig. 9:**
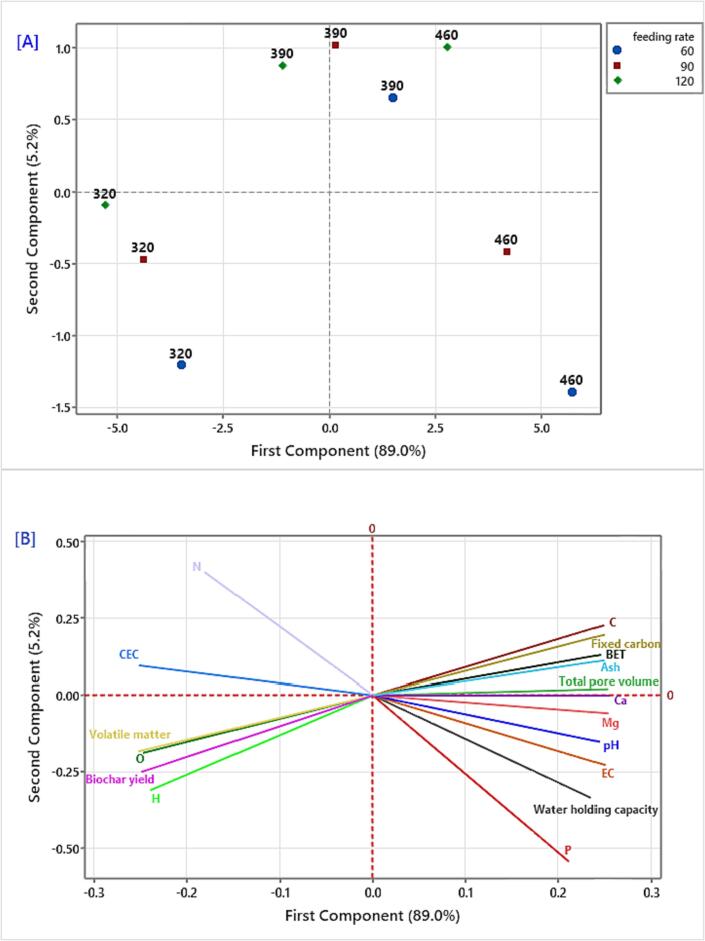
Principal component analysis of DPF biochar subjected to temperature and feeding rate treatments [a] The location of heating treatments and feeding rates, [b] The Location of physicochemical parameters.

Principal Component Analysis (PCA) was employed to analyze the data in order to establish a correlation between biochar samples and physicochemical parameters after the carbonization process. The score plots derived from principal component analysis (PCA) of treated biochar samples after different operation conditions in terms of pyrolysis temperatures and feeding rates are displayed in Fig. 9[a]. The spatial distribution of quality parameters within the space delineated by the first and second principal component analysis (PCA) dimensions is shown in Fig. 9[b]. The combined contribution of principal components 1 and 2 (PC1 and PC2) explained 94.2 % of the observed variability in the biochar samples. Principal Component 1 (PC1) accounted for 89.0 % of the total variation, while Principal Component 2 contributed 5.2 %. PC1 was positively correlated with C, fixed carbon, BET, ash, total pore volume, Ca, Mg, pH, Ec, water hold capacity, and P and negatively with N, CEC, volatile matter, O, biochar yield and H, which showed higher levels biochar samples treated with 390 °C and 460 °C of pyrolysis temperature.

**Fig. 10:**
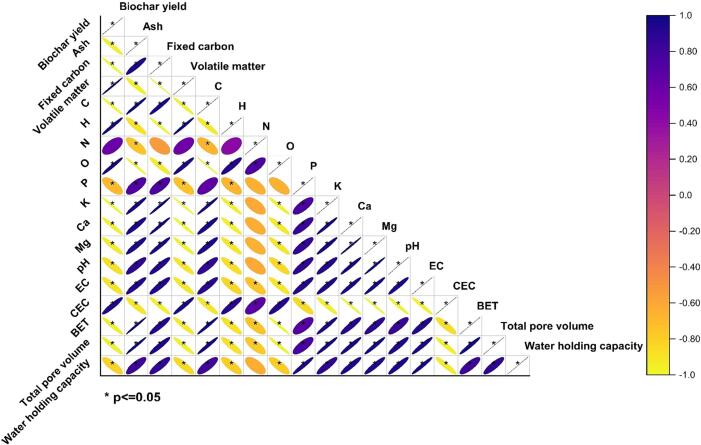
Pearson's correlation analysis between the physicochemical properties of treated biochar under various operation conditions.

Pearson's correlation analysis of the alterations in physicochemical characteristics of treated biochar under various operation conditions of pyrolysis temperatures and feeding rates was presented in Fig. 10. Considering the variances in biochar properties, it was produced under the influence of different pyrolysis temperatures (320, 390, and 460 °C) and feeding rates of 60, 90, and 120 kg/h. The study employed a correlation-based approach utilizing the Pearson coefficient to examine the positive and negative associations among the physiochemical parameters of the treatment process for DPF biochar production. Significant correlations (P ≤0.05) and insignificant relationships (P >0.05) are presented in Fig. 10. The results of the Pearson correlation analysis indicated a statistically significant positive correlation between variable A and the following factors: volatile matter, hydrogen (H), nitrogen (N), oxygen (O), and cation exchange capacity (CEC). Meanwhile, it negatively correlated with ash, fixed carbon, C, K, Ca, Mg, pH, EC, BET, total pore volume, and water hold capacity, which is indicated to lose these properties during the carbonization process.

## Conclusion

The findings indicate that increased pyrolysis temperature decreased biochar yield and volatile matter from date palm fronds biomass. Additionally, a slight increase in biochar yield and volatile matter was observed with an increased feed rate. The highest recorded values of biochar ash content and fixed carbon were achieved when subjecting the material to a pyrolysis temperature of 460 °C and a 60 kg/h feed rate. The results indicated that the increasing temperature increased the nutrient content of P, K, Ca, and Mg. The BET surface area, total pore volume, and water-holding capacity of the biochar produced exhibited an increasing trend with the rise in pyrolysis temperature. However, the feed rate had a slight effect on these properties. The H and O values of the biochar generated exhibited an upward trend with increasing pyrolysis temperature, while a downward trend was observed with an increase in feed rate. A contrary pattern was noted in the values of C and N. The electrical conductivity (EC) and pH levels exhibited an upward trend as the pyrolysis temperature was increased, whereas they displayed a downward trend with an increase in feed rate. Conversely, the cation exchange capacity (CEC) values demonstrated a contrary pattern with no significant variations. Generally, the pyrolysis of date palm fronds at 460 °C and a 60 kg/h production rate resulted in a good biochar of satisfactory physical and chemical characteristics suitable for soil amendment.

## CRediT authorship contribution statement

**Mahmoud Younis:** Software, Resources, Writing – review & editing, Supervision, Funding acquisition. **Hesham A. Farag:** Conceptualization, Validation, Formal analysis, Visualization, Supervision, Project administration. **Abdulla Alhamdan:** Investigation, Resources, Writing – review & editing, Project administration, Funding acquisition. **Galal Aboelasaad:** Methodology, Investigation, Writing – review & editing. **Assem I. Zein El-Abedein:** Validation, Formal analysis, Investigation, Resources, Writing – review & editing, Funding acquisition. **Reham M. Kamel:** Conceptualization, Methodology, Software, Validation, Formal analysis, Investigation, Resources, Data curation, Writing – original draft, Visualization, Supervision, Project administration.

## Declaration of Competing Interest

The authors declare that they have no known competing financial interests or personal relationships that could have appeared to influence the work reported in this paper.

## Data Availability

Data will be made available on request.
